# Student evaluation of medical school curriculum transformation in Iraq

**DOI:** 10.15694/mep.2020.000052.1

**Published:** 2020-03-26

**Authors:** Huda Noori Jawad, Zainab Amir Abd-alnabi, Layla Mohammed Abd-alKadir, Noor Falah Hassan, Zahraa Abbas Mutlaq, Krishna Doshi, Michael Kron, Taghreed K Alhaidari

**Affiliations:** 1 Al Kindy College of Medicine; 2 Medical College of Wisconsin

**Keywords:** Medical Education, Iraq, University of Baghdad, Al Kindy College of Medicine, Curriculum evaluation

## Abstract

This article was migrated. The article was marked as recommended.

**Introduction:** Decades of political and social unrest negatively impacted medical education in Iraq. Recently, new opportunities arose for medical schools to engage international education organizations and the World Health Organization to implement medical school curriculum changes, replacing older discipline-based, teacher-focused systems with a systems-based, student-focused reformed curriculum.

**Methods:** A descriptive, cross sectional quantitative study was designed to survey medical students near the beginning (years 2-3) and at near the end (years 5-6) of their six-year program at the Al Kindy College of Medicine, University of Baghdad, Iraq.

**Results:** A validated questionnaire collected data on thirty-two issues, including student perception of learning, student perception of teachers, academic self-perception and student self-perception. Seven of the thirty-two questions included in this survey resulted in significantly different responses from group 1 (second and third year) vs group 2 (fifth and sixth year) students.

**Conclusions:** This study concluded that the two student groups were significantly different in their awareness of the need for curriculum change, but that student self-perception in both groups was less than ideal at present. In the future, studies are planned to assess student confidence in their professional development, as teaching institutions advance toward broader accreditation and thus opportunities for their students.

## Introduction

Decades of social unrest, magnified by the impact of large internally displaced populations within and surrounding Iraq, the unintended consequences of international sanctions and fluctuating oil prices, has severely and negatively impacted medical education in Iraq ( Simpson, 2017; Kron *et al*., 2019). After the fall of the previous regime in 2003, there were tremendous disruptions to Iraqi society, including the destruction of over 20% of the nation’s hospitals and widespread kidnapping or death of more than 2,000 physicians and medical students (Al-Kalisi, 2010; Al-Kalisi, 2013; Amin and Khoshnaw, 2003; Barnett-Vanas *et al*., 2016; Burnham, Lafta and Doocy, 2009; Jalili, 2007; Tarzi, 2015; Webster, 2009). Unofficial statistics from the Al Kindy College of Medicine (AKCOM), University of Baghdad, indicate that only a small minority of medical graduates have the opportunity to take USMLE step exams because they are not offered in-country and the costs of traveling outside of Iraq for exams is prohibitive. Not surprisingly, the prevalence of post-traumatic stress amongst Iraqi citizens in 2015 was calculated at 11%, particularly affecting women of all ages ( Bradley, 2016; Steel, 2009; WHO, 2019).

The first Iraqi medical school was established in 1927 based on a British model, however by 1988 the entire medical education structure had been disrupted by the Iraq-Iran war. Inrecent years, with leadership from the World Health Organization, the World Federation of Medial Education, and the National Council for Accreditation of Medial Colleges in Iraq, major initiatives have been undertaken to transform the core structure of medical education in all thirty Iraqi medical schools by modifying curriculum content and teaching styles in order to provide students with appropriate learning environments, knowledge and skill sets that will in turn restore excellence and self-confidence as they progress to more independent patient care (WHO, 2016; Yousif, 2007; Hani and Gouda, 2014; Al-Shamsi, 2017).

In 2016, the Iraqi Ministries of Health and Education and AKCOM engaged its administration and curriculum committee to critically review its entire medical school curriculum, and with input from a variety of international accreditation agencies, work to transform a traditional discipline based curriculum and teacher-centric mindset, into a more modern integrated and student centered teaching atmosphere ( Quintero *et al*., 2016; Enns *et al*., 2016; Costly, 2015; Norman and Schmidt, 1992). As in most countries, major transformations in curricula have the potential to at least temporarily, increase anxiety or confusion in the students and faculty, since there is often no other structure with which to gauge the success of new quality improvement measures.

Therefore, to gain information about student perceptions about curriculum change, as well as their general perception of learning, teachers, academic self-perception and social self-perception, a formal survey of these elements was designed, implemented and completed ( Richards, 2000; Stromso, 2004). In the future, this data will be critically important to measure and guide the continued transformation of medical education in Iraq. Understanding student perceptions and responses to new academic pressures in a country experiencing special social-cultural challenges including the displacement of millions of culturally distinct populations, is considered key to appropriate redesign and modification of new integrated curricula that will in turn lead to broader accreditation opportunities for institutions and students ( Al-Hemiary *et al*., 2017; Al-Hilfy, 2015; Lafta *et al*., 2018).

## Methods

A cross sectional descriptive quantitative study was carried out among students in the 2 ^nd^ through 6 ^th^ year of the six-year curriculum of the Al Kindy College of Medicine. Data was collected between December 10, 2016 and May 18, 2017. Responses were collected from 294 students, 159 in group 1 (second and third year students) and 135 in group 2 (fifth and sixth year students). The 32-question survey was self-administered and included two questions about student awareness of the type of curriculum being taught and an additional thirty questions about student perceptions of learning, perceptions of teachers, academic self-perception, perception of atmosphere and social self-perception ( Seneviratne, 2001). This survey was approved by the research ethics committee of the AKCOM.

Collected data were organized, tabulated and analyzed using Statistical Package for Social Sciences (SPSS) for windows, version 17.0, IBM, USA. Descriptive statistics of variable were presented as frequencies and percentages for categorical variables. Comparisons between student groups 1 and 2 were tested by student’s test (Chi Square) for all continuous variables according to categorical variables (stage). P values were calculated as levels of significance and values <0.05 were considered significant.

## Results/Analysis

Seven of the thirty-two questions included in this survey resulted in significantly different responses from group 1 (second and third year) vs group 2 (fifth and sixth year) students [ Table 1]. Fifth- and sixth-year students (group 2) were more aware in general of the types of curriculum in medical school (traditional vs integrated) than second and third year students (group 1). However, the groups were equally aware or unaware of the type of curriculum they were then experiencing. On the topic of student perception of learning, 48.4 % of group 1 agreed that teaching is student-centered compared to only 34.8% of group 2. 68% of all students agreed or somewhat agreed that teaching was too teacher-centered. 92.4% of group 1 students seemed agreeable that teachers were knowledgeable about their assigned subjects compared to 92.9% of group 2, and a minority in each group had the opinion that teachers were not knowledgeable. Group 1 and group 2 students differed on whether their problem-solving skills were being well-developed but group 1 more than group 2 believed that the new curriculum was enhancing their communication and research skills. Group 2 students were more bothered from classroom crowdedness but also were more positive than group 1 in terms of academic self-perception. The overall picture of social-self -perception was less than ideal, with 53.4% of all students (group 1 plus group 2) having an unfavorable view of existing support systems, specifically those for students feeling stressed by any combination of academic or external pressures.

**Table 1.** Survey administered to 294 students at the Al Kindy College of Medicine, University of Baghdad, Iraq.

Questions	Responses				P value
**1. How many types of curriculum are in our college?**	**Don’t know**	**One**	**Two**	**Three**	
	25.8%	5.0%	64.3%	4.8%	0.03*
**2. What type of curriculum are you engaged with?**	**Traditional**	**Integrated**	**Neither**	**Both**	
	44.1%	46.3%	4.8%	4.8%	0.00

Student perception of learning	Agree	Somewhat agree	Disagree	
**3. The teaching is student-centered**	42.2%	39.8%	18%	0.011*
**4. The teaching is well focused**	17.7%	57.8%	24.5%	0.131
**5. The teaching helps to develop my self-confidence**	27.9%	41.1%	31%	0.036*
**6. I am clear about the learning objectives of modules and lectures**	25.9%	53.0%	21.1%	0.089
**7. The teaching is too teacher-centered**	22.4%	42.5%	35.1%	0.047*
**8. Technology is used to deliver information in the classroom**	38.1%	42.5%	19.4%	0.130
**9. Crowded classrooms hamper the use of teaching methods**	48.0%	27.9%	24.1%	0.095
**10. Students are actively involved in planning learning and choices**	23.1%	40.8%	36.1%	0.811
**Student perceptions of teachers**				
**11. Teachers are knowledgeable**	46.3%	46.6%	7.1%	0.044*
**12. Teachers have good communication skills**	18.4%	58.5%	23.1%	0.132
**13. Teachers give clear examples**	28.6%	53.7%	17.7%	0.081
**14. Teachers are well prepared for class**	26.8%	54.8%	18.4%	0.242
**Student academic self-perception**				
**15. I am confident that I will pass this year**	53.7%	38.1%	8.2%%	0.403
**16. I feel I am being well prepared for my profession**	26.2%	48.6%	25.2%	0.088
**17. I can memorize all that I need to**	11.2%	51.0%	37.8%	0.631
**18. My problem-solving skills are being well developed**	18.7%	53.4%	27.9%	0.046*
**Student perception of atmosphere**				
**19. Atmosphere is relaxed during labs**	28.9%	42.9%	28.2%	0.426
**20. Atmosphere is relaxed during lectures**	26.2%	49.3%	24.4%	0.270
**21. Atmosphere is relaxed during seminars/tutorials**	31%	40.1%	28.9%	0.356
**22. Cheating is a problem in this school**	33.0%	29.6%	37.4%	0.964
**23. I can ask questions when I need to**	31.6%	41.2%	27.2%	0.250
**Student social self-perception**				
**24.There is a good support system for students who get stressed**	17.3%	29.3%	53.4%	0.820
**25. I am too tired to enjoy lectures**	50.3%	36.1%	13.6%	0.431
**26. My curriculum enhances patient management skills**	69.7%		30.3%	0.135
**27. My curriculum enhances communication skills**	75.5%		24.5%	0.031*
**28. My curriculum enhances research skills**	74.5%		25.5%	0.042*
**29. My curriculum enhances ability to work in health teams**	67.0%		33.0%	0.267
**30. My curriculum enhances independent learning skills**	68.0%		32.0%	0.000
**31. The curriculum is more time consuming**	62.0%		38.0%	0.617
**32. The curriculum is more stressful**	71.1%		28.9%	0.994

P values refer to differences between the two student groups surveyed. Group 1: 159 2nd and 3rd year students. Group 2: 135 5th and 6th year students. Asterisks (*) indicate statistically significant differences between the two groups. Percentages indicate the total responses of group 1 plus group 2.

## Discussion

This survey represents findings from the first medical student evaluation of a major curriculum change in more than a decade at the Al Kindy College of Medicine. As part of new student orientation, discussions and documents regarding curriculum structure is presented. However, when new curriculum was being implemented, there was concern that students in their 5 ^th^ and sixth years of medical school might be confused or upset by the major changes. Ideally, measures of the success of the new curriculum would involve comparison of variations in knowledge between Iraqi schools that have or have not implemented new teaching methods. At present, there is no internal country wide examination that is administered to students at all 30 Iraqi medical schools.

Correlation between perception of the learning environment and performance (academic success) needs to be studied. In the spirit of new teaching methodology, continuous formative evaluation of the curriculum is required through teacher and student feedback to identify and solve problems to foster a more inclusive and cooperative learning environment. During the transition from discipline based- to integrated curriculum, there still may be too much of a dependence on formal lectures that do not fully shift the balance toward student-driven learning (more beneficial in the long term.) From the faculty perspective, such a major transition to integrated or problem based learning will still require ongoing encouragement and non-threatening positive feedback that is aimed at further improving the student-teacher relationship.

Integration of curriculum with early clinical exposures is intended to reduce the number of information “silos” and should help to avoid redundancy that leads to information overload. From the student perspective, the broadly beneficial effects of integrated learning should be all the following: improved motivation and satisfaction, professional socialization, enhanced self-reflection and self-appraisal, reinforced learning, better preparation for life-long learning, heightened relevance of new materials, facilitated curriculum updates and review and better clinical reflections on basic science principles. Changes in Iraqi curricula are intended to highlight and reinforce the American Association of Medical Colleges goals for medical education, as well as the World Health Organizations concept of the “five-star doctor for the twenty-first century: a physician should be a care provider, decision maker, communicator, manager and leader, community minded and cognizant of relevant up-to-date research ( Boelen, 1994; Cooke, 2006).

Given the history of societal upheaval in Iraq, “academic self-concept” and social-self-concept are sometimes difficult to separate, yet they lie at the heart of long term efforts to maintain self confidence in a medical education system that no longer will allow itself to fall behind the times. Academic self-concept is the perception that a student has about his/her own academic abilities, and is one of the most important variables in the academic world, because of its influence on learning. Social self-perception is defined as the individual’s belief about himself or herself, including the person’s attributes. Encouraging movement toward these goals is exemplified by forward thinking and optimistic AKCOM student’s enthusiastic endorsement of the Universal Declaration of Human Rights ( SCORP, 2016).

## Conclusion

This study concluded that the two student groups were significantly different in their responses to seven of the thirty-two survey questions. They differed in their awareness of the need for curriculum change, and student self-perception in both groups was less than ideal at present. Group one and group two students were significantly different in their understanding of curriculum design, learning self-perceptions, teaching structure and knowledge in teacher effectiveness. In the future, studies are planned to assess student confidence in their professional development, as teaching institutions advance toward broader accreditation and thus opportunities for their students.

## Take Home Messages

• Medical students in Iraq face complex academic and socio-cultural challenges, some of which are the result of immense societal upheaval over the past several decades.
• Recent transformative changes in medical school curriculum and teaching styles pose a new challenge to students contemplating medical school as well as for those already studying within the established six-year medical structure.
• The reasons for sweeping curriculum changes being implemented by Iraqi schools are not uniformly understood by current students.
• There is cautious optimism that new approaches will lead to improved student and teacher performance, and greater opportunities for school accreditation.

## Notes On Contributors

**Huda Noori Jawad** is a medical student in good standing presently enrolled at the Al Kindy College of Medicine, University of Baghdad who participated in this survery as part of a student led project. All students equally participated in the design, collection and interpretation of data.

**Zainab Amir Abd-alnabi** is a medical student in good standing presently enrolled at the Al Kindy College of Medicine, University of Baghdad who participated in this survery as part of a student led project. All students equally participated in the design, collection and interpretation of data.

**Layla Mohammed Abd-alKadir** is a medical student in good standing presently enrolled at the Al Kindy College of Medicine, University of Baghdad who participated in this survery as part of a student led project. All students equally participated in the design, collection and interpretation of data.

**Noor Falah Hassan** is a medical student in good standing presently enrolled at the Al Kindy College of Medicine, University of Baghdad who participated in this survery as part of a student led project. All students equally participated in the design, collection and interpretation of data.

**Zahraa Abbas Mutlaq** is a medical student in good standing presently enrolled at the Al Kindy College of Medicine, University of Baghdad who who participated in this survery as part of a student led project. All students equally participated in the design, collection and interpretation of data.

**Krishna Doshi** is a third year medical student at the Medical College of Wisconsin who focused on medical education in Iraq for her Global Health Pathway concentration area. Her contributions included data organization and manuscript formatting issues.

**Taghreed Alhaidari** is professor of Obstetrics and Gynecology at the Al Kindy College of Medicine, University of Baghdad. She is the faculty supervisor of all students for this project. In addition, she contributed in terms of study design, statistical analysis and data interpretation. ORCID iD: https://orcid.org/0000-0001-8494-8760

**Michael A. Kron** is a professor of Medicine, Medical College of Wisconsin, Milwawukee, Wisconsin USA. He is a medical education collaborator who played a key role in assisting Al Kindy College of Medicine leadership in its curriculum reform process. In addition, he assisted with formatting, data analysis and writing of this manuscript. ORCID iD: https://orcid.org/0000-0002-8240-5209

## Declarations

The author has declared that there are no conflicts of interest.

## Ethics Statement

The institutional research committee of the Al Kindy College of Medicine, University of Baghdad reviewed and approved this curriculum evaluation study on 11 August 2016, document reference number 142.

## External Funding

This article has not had any External Funding

## References

[ref1] Al-Shamsi M. (2017) Medical education in Iraq: Issues and challenges. International Journal of Medical Education. 8, pp.88–90. 10.5116/ijme.58b1.c927 28285276 PMC5357542

[ref2] Al-Khalisi N. (2013) The Iraqi medical brain-drain: A cross-sectional study. International Journal of Health Services. 43, pp.363–378. 10.2190/HS.43.2.j 23821910

[ref3] Al-Hemiary N. Al-Nuaimi A.S. Al-Saffar Hl and Randall I. (2017) Why people apply to medical school in Iraq? Journal of Medical Education and Curriculum Development. 4, pp1–5. 10.1177/2382120517726997 PMC5736275 29349341

[ref4] Al-Hilfi T. alhaidari T. Mohammed M. Abdul-Kareem A. (2015) Reviewing of curriculum analysis in medical education in Iraq. New Iraqi Medical Journal. 1pp.62–66.

[ref5] Al-Khalisi N. (2010) The perils of being a doctor in Baghdad. British Medical Journal. 341:c4043. 10.1136/bmj.c4043 20667953

[ref6] Amin N.M. and Khoshnaw M.Q. (2003) Medical education and training in Iraq. Lancet. 362:1326. 10.1016/s0140-6736(03)14580-1 14579817

[ref7] Barnett-Vanes A. Hassounah S. Shawki M. Ismail O. (2016) Impact of conflict on medical education: A cross-sectional survey of students and institutions in Iraq. British Medical Journal Open. 6(2): e010460. 10.1136/bmjopen-2015-010460 PMC4762136 26883241

[ref8] Boelen C. (1994) Frontline doctors of tomorrow. World Health. 47(5) pp.4–5. World Health Organization. https://apps.who.int/iris/handle/10665/328599( Accessed: 11 March 2020).

[ref9] Bradley M . (2016) Iraq’s doctors face threats of violence. Wall Street Journal. https://www.wsj.com/articles/iraqs-doctors-face-threats-of-violence-1462145946( Accessed: 27 February 2020).

[ref10] Burnham G.M. Lafta R. and Doocy S. (2009) Doctors leaving 12 tertiary hospitals in Iraq, 2004-2007. Social Science Medicine. 69(2), pp.172–177. 10.1016/j.socscimed.2009.05.021 19501443

[ref11] Cooke M. Irby D.M. Sullivan W. and Ludmerer K.M. (2006) American medical education 100 years after the Flexner Report. England Journal of Medicine. 3, pp.1339–1334. 10.1056/NEJMra055445 17005951

[ref12] Costley K.C. (2015) Research Supporting Integrated Curriculum: Evidence for Using This Method of Instruction in Public School Classroom. https://files.eric.ed.gov/fulltext/ed552916.pdf( Accessed: 27 February 2020).

[ref13] Enns S.C. Perotta B. Paro H.B. Gannam S. (2016) Medical students’ perception of their educational environment and quality of life: is there a positive association? Academic Medicine. 91(3), pp.409–417. https://doi.org./10.1097/ACM.0000000000000952 26556293 10.1097/ACM.0000000000000952

[ref14] Hani S. and Gouda E.M. (2014) Curriculum Integration in Medical Education, IbnSina National College for Medical Studies. Jeddah, Saudi Arabia. 2014. April 1. https://www.esciencecentral.org/journals/curriculum-integration-in-medical-education-a-theoretical-review-ipr.1000113.php?aid=25085( Accessed: 27 February 2020).

[ref15] Jalili I. (2007) Iraqi academics and doctors: Innocent victims of a wider geopolitical struggle. https://citeseerx.ist.psu.edu/view doc/download?doi=10.1.1.551.7612&rep=rep1&type=pdf( Accessed: 27 February 2020).

[ref16] Jawad H. (2017) Orientation documents for Al Kindy medical school students. Al-Kindy College of Medicine, University of Baghdad, Baghdad, Iraq.

[ref17] Kron M. Roenius M. Alqortasi M.A. Alhaidari T. (2019). Academic Medicine and Science Diplomacy: Medical Education in Iraq. Academic Medicine. 94(12), pp.1884–1890. 10.1097/ACM.0000000000002918 31365401

[ref18] Lafta R. Al-Ani W. Dhiaa S. Cherewick M. (2018) Perceptions, experiences and expectations of Iraqi medical students. BMC Medical Education. 8, pp.53–58. 10.1186/s12909-018-1156-8 PMC5870482 29587726

[ref19] Norman G.R. and Schmidt H.G. (1992) The psychological basis of problem-based learning: A review of the evidence. Academic Medicine. 67, pp.557–65. 10.1097/00001888-199209000-00002 1520409

[ref20] Quintero G. Vergel J. Arredondo M. Ariza M. (2016) Integrated Medical Curriculum: Advantages and Disadvantages. Journal of Medical Education and Curriculum Development. 3, pp.133–137. 10.4137/JMECD.S18920 PMC5736212 29349303

[ref21] Richards L.J. and Wall S.N. (2000) Iraqi medical education under the intellectual embargo. Lancet. 355, pp.1093–1094. 10.1016/S0140-6736(00)02049-3 10744107

[ref22] SCORP (2016) Human Rights & Peace. International day of human rights/Al Kindy College of Medicine. https://youtu.be/qJXIiPsaiT4( Accessed: 27 February 2020).

[ref23] Seneviratne R.D. Samarasekera D.D. Karunathilake I.M. and Ponnamperuma G.G. (2001) Students’ perception of problem-based learning in the medical curriculum of the faculty of medicine, University of Colombo. Ann Acad Med Singapore. 30, pp.379–381. 11503544

[ref24] Simpson B. (2017) Rebuilding medical education in Iraq. Global Health Now. Johns Hopkins Bloomberg School of Public Health. https://www.globalhealthnow.org/2017-11/rebuilding-medical-education-iraq( Accessed: 27 February 2020).

[ref25] Steel Z. Chey T. Silove D. Marnane C. (2009) Association of torture and other potentially traumatic events with mental health outcomes among populations exposed to mass conflict and displacement: A systematic review and meta-analysis. Journal of the American Medical Association. 302, pp.537–549. 10.1001/jama.2009.1132 19654388

[ref26] Strømsø H.I. Grøttum P. and Hofgaard Lycke K. (2004) Changes in student approaches to learning with the introduction of computer supported. Medical Education. 38(4), pp.390–398. 10.1046/j.1365-2923.2004.01786.x 15025640

[ref27] Tarzi N. (2015) Medical doctors in Iraq: Vilified, hunted and killed. Global Research, Centre For Research on Globalization website. http://www.globalresearch.ca/medical-doctorsin-iraq-vilified-hunted-and-killed/5496148( Accessed: 27 February 2020).

[ref28] Webster P. (2009) Medical faculties decimated by violence in Iraq. Canadian Medical Association Journal. 181, pp.576–578. 10.1503/cmaj.109-3035 19786474 PMC2764751

[ref29] World Health Organization Regional Office for the Eastern Mediterranean . (2016) Summary report on the workshop on accreditation of medical education in Iraq: Towards excellence in medical education and health care. http://applications.emro.who.int/docs/( Accessed: 27 February 2020).

[ref30] World Health Organization Regional Office for the Eastern Mediterranean . (2019) WHO health emergencies: Displaced populations. http://www.emro.who.int/eha/displaced-populations/index.html( Accessed: 27 February 2020).

[ref31] Yousif T.K. (2007) Towards quality and accreditation in health professions education in Iraq. Middle East Journal of Family Medicine. 5, pp.3–7.

