# Supplementary material for: Student evaluation of medical school curriculum transformation in Iraq
Source: MedEdPublish (2016). 2020 Mar 26;9:52. [Version 1] doi: 10.15694/mep.2020.000052.1 (PMC10697557; doi:10.15694/mep.2020.000052.1)
Supplement: Peer review history

# Reviewer report: Reviewer response for version 1

**DOI:** 10.21956/mep.20011.r30514

**Published:** 2020-03-30

**Authors:** Deb Halder

This review has been migrated. The reviewer awarded 5 stars out of 5 The strongest parts of the Paper:The use of cross-sectional study has tended to validate the study as universal trait for the Iraq medical school scenario. The descriptive analysis has fostered the results more readable and the comparative study between two guided groups of students has made the presented data adaptable and adoptable.The abstract can present the paper in a nut shell, introduction has been able to draw the point of existing problem dealt in the paper, the rationale of the study along with suitable references. The references are up to date.The issues of ConcernThere are some critical issues of this paper that needs to be addressed. First of all, the sampling procedure does not make any clarity and why this 294 respondents were chosen and whether they were approached ethically cannot be understood.On the page. 45, there is the insertion of formative and summative assessment. But these two terms have not been inserted and clarified at the expositional part of the paper. More reviews on literature are required to defend these sorts of terms and tools.The in- text citation of Bolen, 1994 and Cooke, 2006 with the indication of American Association of Medical College goals cannot be realized with the former conception in the paragraph. It has made disjointed divisions in this paragraph (p.45).The definition of ‘Academic self – concept and Social self – concept’ in the discussion part has not been provided with references which fails to claim reliability of these definitions. The ‘discussion’ aims at analyzing the result critically and what the result really predicts. But this paper has amalgamated discussion with recommendation which is a violation of construction of an academic paper as this sort of amalgamation loses the beauty of stylistics. At p.45, the directive as ‘ continuous formative evaluation of curriculum is required through teacher and student feedback’ is similar to recommendation which has been added in the discussion part of the paper. The paper could have added recommendation. Again, this reference has not been cited properly “Jawad H, Abd-alnabi Z, Abd-alKadir L, Hassan N, Mutlaq Z, Doshi K, Kron M, Alhaidari T. MedEdPublish https://doi.org/10.15694/mep.2020.000052.1” as it misses year of publication

Reviewer Expertise:

NA

No decision status is available

# Reviewer report: Reviewer response for version 1

**DOI:** 10.21956/mep.20011.r30511

**Published:** 2020-03-29

**Authors:** faris khazaal

**Affiliations:** 1 University of baghdad

This review has been migrated. The reviewer awarded 4 stars out of 5 In each type of undergraduate medical learning curriculum , there is cons and pros, the most important is how to apply so that we can reach the goalsi think there is always a need to show the students and to test the aims of there education curriculum what ever the results of this study it should explain the differences between the answers between juniors and seniors students and to have recommendations to overcome gaps in learning process.

Reviewer Expertise:

NA

No decision status is available

# Reviewer report: Reviewer response for version 1

**DOI:** 10.21956/mep.20011.r30515

**Published:** 2020-03-28

**Authors:** Trudie Roberts

**Affiliations:** 1 Leeds Institute of Medical Education

This review has been migrated. The reviewer awarded 3 stars out of 5 Firstly my congratulations to colleagues in Iraq for writing up and sharing their study. Given the issues and challenges that Iraq has come through their commitment is humbling. As others have commented it would have been good to see a copy of the questionnaire appended to this submission so we could have viewed the questions, together with some information about the types of students enrolled. For example are they direct entry from school, are they all single gender, are they still likely to be living at home with parents and a little more about the medical school culture.I am intrigued to know more about your plans to address the issues you have identified and how plan to continue to monitor student development afterwards.

Reviewer Expertise:

NA

No decision status is available

# Reviewer report: Reviewer response for version 1

**DOI:** 10.21956/mep.20011.r30513

**Published:** 2020-03-28

**Authors:** P Ravi Shankar

**Affiliations:** 1 American International Medical University

This review has been migrated. The reviewer awarded 4 stars out of 5 This is an interesting manuscript about student perception regarding changes in the medical curriculum in a medical school in Iraq. Student evaluation of the curriculum has been presented. Many authors of the paper are medical students. The study has been well written and presented. Many parameters measured in the present study seem to have similarities with those in the Dundee Ready Education Environment Measure (DREEM). The overall classification scheme also seems to have similarities. I would be interested in knowing how the questionnaire was developed by the authors. How was it validated? In which language was it administered? What was the overall response rate? I am also interested ion knowing greater details about the curricular modifications carried out in Iraq. From the paper I understand it is being implemented at all medical schools in the country. The international collaboration between students and faculty is another strength of the paper. The paper would be of interest to a broad group of medical educators.

Reviewer Expertise:

NA

No decision status is available

# Reviewer report: Reviewer response for version 1

**DOI:** 10.21956/mep.20011.r30512

**Published:** 2020-03-27

**Authors:** Judy McKimm

**Affiliations:** 1 Swansea University

This review has been migrated. The reviewer awarded 3 stars out of 5 Dear allmany thanks for submitting this article. I was really interested to hear about the former and current situation in Iraq, and I think many people would be keen to hear how you have been addressing such immense challenges. I think that this could benefit from some strengthening in a couple of areas. First, it needs to clearer how the questionnaire was designed and what you were really trying to find out, i.e. what was the intended outcome from this (not in terms of what you would find out, but more what you would then do with it)? Secondly, I would like the discussion/conclusions to be stronger, again about the 'so what?' elements. What specifically will you do with what you have found, why do the differences matter? what will you put in place to address the issues etc?I hope that is helpful, well done for doing this in the midst of all your struggles.

Reviewer Expertise:

NA

No decision status is available
